# Prevalence and Factors Associated with Postpartum Depression in Primary Healthcare Centres in Yangon, Myanmar

**DOI:** 10.21315/mjms2021.28.4.8

**Published:** 2021-08-26

**Authors:** Theigi Myo, Seo Ah Hong, Bang-on Thepthien, Nate Hongkrailert

**Affiliations:** ASEAN Institute for Health Development, Mahidol University, Nakhon Pathom, Thailand

**Keywords:** prevalence, postpartum depression, Myanmar

## Abstract

**Background:**

Postpartum depression (PPD) can have serious consequences on both the mother and infant. Despite the higher prevalence, there are limited numbers of studies on PPD in low-and middle-income countries, like Myanmar. This study aimed to explore the prevalence and associated factors of PPD in primary healthcare settings in Myanmar.

**Methods:**

This cross-sectional online study was conducted with 220 mothers under 6 months postpartum in April–May 2020 and who registered in public health centres in Kungyangone Township, Yangon, Myanmar. The postpartum depression was measured with the Edinburgh postpartum depression scale (EPDS, ≥ 13 scores). Independent variables included sociodemographic factors, obstetric and infant factors, psychosocial factors (social support and social media usage), health services utilisation and accessibility factors. Chi-square tests and multiple logistic regression were performed.

**Results:**

Overall prevalence of depressive symptoms in 220 women under 6 months postpartum was 31.8% (95% confidence interval [CI]: 25.9, 37.3). In multiple logistic regression, unplanned pregnancy (adjusted odds ratio [AOR]: 2.946), less than four times antenatal care (ANC) visits (AOR: 2.518), travel time more than 1 h to reach health centres (AOR: 3.068) and birth interval more than 5 years (AOR: 4.594) were more likely to be associated with PPD, while preterm delivery (AOR: 0.091) was inversely associated.

**Conclusion:**

This study showed the relatively high prevalence of PPD and the strong association with preterm delivery, pregnancy intention, breastfeeding status, birth interval as well as frequency of ANC received and travel time to health centre. It may suggest that maternal mental health services should be integrated with existing maternal and child health (MCH) services for early detection and prevention of depression symptoms with promotion of MCH services utilisation and improved accessibility among mothers in primary healthcare setting.

## Introduction

Postpartum depression (PPD) is a frequent health problem for women during the postpartum period and a major contributor to maternal morbidity and mortality. It has adverse effects on the mother’s well-being and socioemotional and cognitive development of children (1, 2). Although it is a treatable medical disease, it still remained highly under-recognised and undertreated in maternal and child health (MCH) programmes, particularly in low and middle-income countries (LMICs) (3). It is estimated that 7 in 10 women hide or downplay their symptoms without understanding, support and treatment (2). Globally, the prevalence of PPD among mothers ranges from 0.5%–60.8% (4). Most studies on PPD were conducted in high-income countries (5), although the prevalence is reported to be higher in LMICs than in high-income countries (6). Furthermore, since the prevalence is highly variable between populations and its manifestations may vary across cultures (7), future studies should be conducted to understand the nature of women’s PPD experience across different cultures in LMICs. In Myanmar, the prevalence was reported as 19.4% among mothers within 6 months after delivery in a urban area of a township in Shan State (8) and 8.3% of migrant labour and refugee women on the Thai-Myanmar border during perinatal period to be reported as moderate-severe depression (9). Nonetheless, there are limited studies on PPD in LMICs including Myanmar.

To design effective interventions, it is critical that factors related to PPD be understood. Associated factors of PPD are multifactorial and they were included from literature review in our study. Socioeconomic disadvantages, such as unemployment, low income and education have been associated with PPD (6, 10). A review study showed that cigarette smoking is associated with increased risk of moderate and severe depression and the proportion of alcohol drinking and drug use were higher among postpartum mothers (11). In Myanmar, while a prevalence of cigarette smoking is relatively low among pregnant and breastfeeding women (1.6% and 1.5%, respectively) (12), betel quid chewing, which is a smokeless tobacco, is very popular traditionally and also remains high among pregnant and breastfeeding women (17.6% and 21.9%, respectively). A Taiwanese study showed the risk of adverse birth outcome was higher among betel quid chewing women than the non-users (13). Yet, to our knowledge, there was no study on the association between betel quid chewing and PPD. In terms of obstetric and infant factors, parity (14), mode of delivery (15), pregnancy intention (16), preterm delivery (17), breastfeeding (18), and antenatal depression history (19) were reported to be strong predictors of PPD. Accessibility of health services is one of the important factors associated with PPD (20). Utilisation of maternity care services, such as antenatal care (ANC) (9) and postnatal care (PNC) has influence on women’s depressive symptomology and maternal health outcome (21).

In addition, social support and interpersonal relationships have a substantial impact on the physical and mental health of mothers. These specific populations (husbands, parents, family in law and relatives/friends) are close to mothers as social network in the postpartum period. Online support from social media usage has emerged to replace or extend traditional offline support in recent years. Popularity of social networking sites (e.g. Facebook) has become so pervasive in developing countries. Social media might give psychosocial support to new mothers because they may receive support to deal with new responsibilities and manage physical and mental health conditions by usage of social media such as Facebook (22). Studies are needed to figure out if social support navigated through social media is associated with PPD among postpartum mothers.

The Republic of the Union of Myanmar (23) is located in Southeast Asia. The Myanmar is comprised of more than 100 ethnic groups and had an estimated population of 51.5 million in 2014 (24) and it was classified as a lower middle income country by the World Bank in 2018 (25). Maternal mortality ratio (MMR) was estimated as 250 per 100,000 live births in 2017 (26). According to Myanmar’s demographic and health survey (DHS) of 2015–2016, 58.6% of women had at least four ANC contacts and 68% has national prevalence of PNC utilisation (12). Although, World Health Organization (WHO) encouraged promoting mental health as one of the sustainable development goals (SDGs) (27), there were no standard and specific guidelines for maternal mental healthcare services and it still remain neglected in Myanmar (28).

This cross-sectional study aimed to identify the prevalence of PPD and its associated factors among postpartum mothers in Myanmar. Due to little information on prevalence of mental distress among postpartum women and no routine screening test of depression for mothers in Myanmar, our findings will provide evidence of depression for postnatal women and raise awareness regarding the issues mothers encounter. The findings from this study contribute to achieving the national target of the SDGs in Myanmar related to maternal health and well-being by 2030.

## Methods

This cross-sectional study was conducted in Kungyangone Township, Yangon, Myanmar in April 2020 and May 2020. Prior to undertaking the study, the health staffs of township health department were briefed about the study design and purpose and their verbal approval was obtained. Ethics approval was obtained from the Institutional Review Boards (IRB) of Mahidol University, Thailand (No. 2020/03-115). Approval from local authorities and township health department of Kungyangone Township were taken verbally. Due to the COVID-19 preventive measures of lockdown period, the data was collected with online Google form as web-based survey. The questionnaire link was delivered by respective health staffs to mothers. Participants were assured of confidentiality and were told that they could terminate participation at any time without prejudice. Thereafter, they were requested to fill informed consent before accepting web-based questionnaire link. All data were treated anonymously using study identification numbers.

### Participants

In Kungyangone Township, there are 25 sub-rural health centres (SRHCs) in the rural area and one maternal and child health (MCH) centre in urban area. Ten SRHCs and one urban MCH centre were randomly selected. Postpartum mothers who registered the selected health centres were invited. The inclusion criteria were: i) postpartum mothers who were less than 6 months postpartum; ii) subjects who can understand and read Myanmar language; iii) subjects who are above the age of 18 years and iv) subjects who could access online survey and gave consent. Meanwhile, postpartum mothers who were not resident of the study area during the survey period and who were not able to access online and read the questions were excluded.

### Sample Size

The required sample size was 184 with 95% confidence interval (CI) with 5% error of allowance and the prevalence of PPD in a Myanmar study (19.4%) (8). The sample size was 184 using the above formula. The sample size was increased to 220 which accounts for 20% probable non-response.

### Measures of Variables

The PPD, the outcome variable was assessed with the Edinburgh postnatal depression scale (EPDS) which is performed well in community based survey for identifying the depression and was translated and validated in many countries (29). It is a 10-item self-reported questionnaire in which women are asked to rate how they have felt in the last 7 days and each item has 4 Likert scales (0–3 score), resulting in total score range of 0–30. The cutoff point (≥ 13) was used to define mothers with PPD.

Based on the literature review, variables were employed to predict the outcome variable in the study. Sociodemographic variables including mother’s current age, marital status, religion, education and occupation, monthly family income, type of family, residence, and substance use (alcohol drinking, smoking and betel chewing) were collected. Obstetric and infant related factors involved gravida, parity, number of living children, preterm delivery and mode of delivery, pregnancy intention, experiencing health problems (mother and infant), infant’s sex and age, baby gender preference, low birth weight, birth order and interval, birth place, infant feeding practice (breastfeeding pattern and timing of supplementary feeding), and then an antenatal depression history.

The postpartum social support questionnaire (PSSQ) (30) was used to measure the social support assessment of respondents. This questionnaire consists of 33 items to be ranked by participants on a 7-point Likert-type scale, indicating their level of agreement or disagreement. It was designed to assess: i) the major aspects of support (emotional, information and instrumental) ii) the source of the support (husband, parent, family in laws and relatives/friends) and iii) aspects of support specifically related to the demands of childcare (e.g. help with childcare, baby-sitting). The survey has previously been subdivided into four subscales: i) partner; ii) parents; iii) partner’s parents and iv) other friends and relatives. Scores for each subscale were calculated for each participant and the scores were catergorised into tertiles (three equal/proportion). The lowest tertile of scores was identified as low social support. The PSSQ was validated in low-income, African American mothers with Cronbach’s alpha coefficients for each subscale ranging from 0.90–0.96 (31) and in current study, ranging from 0.91–0.97.

In term of social media usage, the questions were adopted and modified from a previous study (32). Questions on social media use for seeking health information and for gaining emotional support were asked with “Do you use social media to get any solution and suggestions for health-related concern? (Yes/No)” and “Do you use social media to get emotional support for stress and depression? (Yes/No),” respectively. Mothers’ experiences of feeling more or less depressed by using social media were asked with two questions, “Did you feel that social media usage can increase your stress and depressive symptoms? “Did you feel that social media usage can decrease your stress and depressive symptoms?” with answer options (Yes/No/Not sure), followed by two questions about reasons, “What are the causes of your depression decreasing/increasing due to the usage of social media site(s)?” Furthermore, a question “Will you recommend social media usage by new mothers? (Yes/No/Not Sure)” was asked.

Health service related factors comprised two parts; health service accessibility (mode of travel, time, cost and perception), ANC/PNC utilisation in terms of timing, frequency and status of completion (completed or not). ‘Completed ANC’ means at least four times ANC visits before delivery and getting four times PNC within 42 days according to WHO guideline (within 24 h, 3th–4th day, 6th–7th day and 42nd day) after delivery was labeled as ‘completed PNC’.

### Data Analysis

Statistical Package for the Social Sciences (SPSS) version 21 was used to carry out statistical analyses. Descriptive statistics were used to analyse frequency and percent/proportion of all variables. Bivariate associations between dependent variable (PPD) and independent factors were assessed using Chi-square test. Those independent variables with *P*-value < 0.1 in the bivariate analyses were employed into final multiple logistic regression using backward method to identify predictors and strength of association with PPD and statistical significance was considered with *P*-value < 0.05.

## Results

Among 220 postnatal mothers, 31.8% (95% CI: 25.9, 37.3) of them were found to have PPD. In Table 1, around half of mothers were 25–35 years old and had education with above middle school completion and had monthly family income less than 200,000 Kyats (USD147). Around one-third was currently working and two-third were housewives. Over 70% of mothers lived in rural areas and 62.7% lived in a nuclear family. Those reporting drinking alcohol and betel quid chewing were 15.5% and 23.2%, respectively, while smokers were only 5.5%. In the bivariate analysis, marital status of mothers (*P* = 0.008), monthly family income (*P* = 0.021) and, smoking (*P* = 0.008) and betel chewing (*P* = 0.001) were associated with PPD.

In Table 2, over 50% of mothers had primigravida, primiparous and having one child and caesarean delivery. Around 10% experienced preterm delivery and antenatal depression history. The mothers used for the study reported that 68.6% of total birth was delivered at government health institutions and 78.2% breastfed their child and 55.5% introduced supplementary foods before 6 months. In the bivariate analyses, gravida (*P* = 0.003), parity (*P* = 0.008), number of living children (*P* = 0.010), preterm delivery (*P* = 0.021), antenatal depression history (*P* = 0.006), birth order (*P* = 0.005) and birth place (*P* = 0.040), mode of delivery (*P* = 0.028), pregnancy intention (*P* < 0.0001), breastfeeding (*P* = 0.007) and supplementary feeding (*P* = 0.007) were significantly associated with PPD.

Table 3 shows the distribution of health centre utilisation and accessibility as well as its association with PPD. About 80% of mothers completed their ANC visits and 95.9% benefitted from PNC services at least once especially within 24 hours of delivery, but only 3.2% completely received PNC services. Travel time to health center (*P* < 0.0001), frequency of ANC received (*P* = 0.003) and PNC within 24 h of delivery (*P* = 0.050) were associated with PPD.

In Table 4, over 80% of participants used social media daily and reported to gain health information via social media and 44.5% reported to gain emotional support from social media usage. Husband (*P* < 0.001) and parent (*P* = 0.003) support among social support subgroups, and frequency of social media use (*P* = 0.003), its usage for health information (*P* = 0.014) and perception that social media decrease stress and depressive symptoms (*P* = 0.006) were associated with PPD.

**Figure 1:**
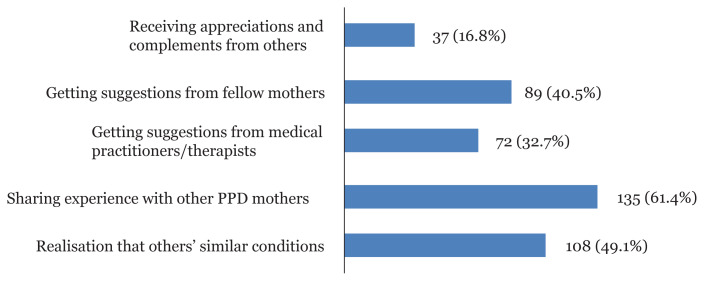
Reasons as to why social media decreases stress and depressive symptoms

**Figure 2:**
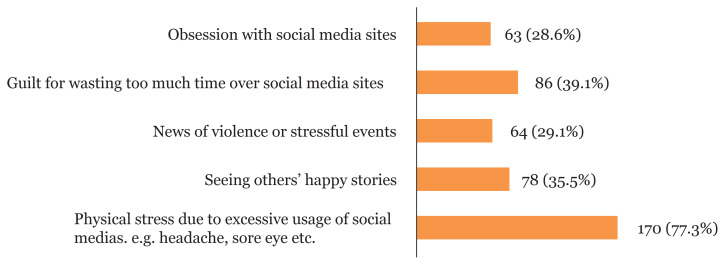
Reasons as to why social media increases stress and depressive symptoms

In Figure 1, the reasons why social media decreases stress and depressive symptoms were sharing experience with other PPD mothers (61.4%), followed by other similar conditions (49.1%), getting suggestions from fellow mothers (40.5%), getting suggestions from medical practitioners/therapists (32.7%) and receiving appreciations and complements from others (16.8%). The reasons why social media increases stress and depressive symptoms (Figure 2) were physical stress due to excessive usage of social media (77.3%), followed by feeling guilty for wasting too much time on social media (39.1%), seeing others’ happy stories (35.5%), news of violence or stressful events (29.1%), and obsession with social media (28.6%).

In the multivariate model (Table 5), unplanned pregnancy (AOR: 2.946, 95% CI:1.301, 6.670), less than four times ANC visits (AOR: 2.518; 95% CI: 1.027, 6.169), travel time more than one hour to reach health centres (AOR: 3.068; 95% CI: 1.178, 7.990) and birth interval more than 5 years (AOR: 4.594; 95% CI: 1.637, 12.891) were more likely to be associated with PPD, while preterm delivery (AOR: 0.091; 95% CI: 0.013, 0.650) was inversely associated with PPD.

## Discussion

### Prevalence of Postpartum Depression

This study showed 31.8% of 220 postnatal women who were on 6 months postpartum in Kungyangone Township, Yangon had PPD. The prevalence was reported to be 39.4% in Bangladesh (15) and 30% in Nepal (33). Compared to the previous study in Shan State, Myanmar (19.4%) (8), our study showed relatively 1.5 times higher prevalence of PPD. The great disparity might be due to some methodological differences. With use of online survey, our subjects had higher education and included both urban and rural areas, while the previous study included only urban areas. Furthermore, despite higher cut-off points of 13, compared to 12 in the previous study, the prevalence in our study was higher. It may be partly due to the worries and stress of mothers as the study period was during the lockdown period of COVID-19 pandemic. The unprecedented situation and during the lockdown period in Myanmar, high loads of domestic chores, more family conflict, low family support/relatives/friends and limited MCH services such as monthly immunisations and postnatal follow-up visits may have put mothers in situation of greater psychological vulnerability and heightened the risk of PPD (10). Although, this study may not represent the prevalence of postpartum mothers under normal circumstances, to our knowledge, this study has a very important novelty that it is the first study to measure the prevalence of PPD during the COVID-19 lockdown period in Myanmar.

### Associations with Sociodemographic and Substance Use Factors

Although marital status and monthly household income were significantly associated with PPD in the bivariate analyses, none of sociodemographic factors remained significant in multivariate analyses in our study. In terms of substance use, smoking and betel quid chewing were not strong predictors of PPD in the multivariate model, but the prevalence of PPD among the mothers who smoked and chewed betel quid was high (66.7% and 51%, respectively). Thus, knowledge about the effect of substance use on maternal wellbeing and safe motherhood should be taught by healthcare providers during ANC and PNC visits.

### Associations with Obstetric and Infant Factors

This study shows that unintended pregnancy is pretty high (44.5%). It may lead to a wide range of health risks for the mother and child, such as illness and death. The unintended pregnancy was associated with PPD in agreement with studies from Bangladesh (34) and Ethiopia (35), whereas another report from Nepal (33) did not find evidence for the same association. The positive association shows that unplanned pregnancy could lead to negative emotional reaction and increase psychosocial stress, and risk of having depression. In addition, our study showed that those with birth interval < 2 years was 68.2% and those with birth interval > 5 years was 11.4%. The wide birth interval was more likely to have PPD, inconsistent with the findings of one systematic review in which short birth interval result in increased risk of postpartum stress, unstable lifestyle and inadequate use of health care services (36). Although, family planning services and contraceptive usage coverage had increased in Myanmar, an unmet need of family planning was found and unplanned pregnancy is still common (12). Thus, ensuring adequate supply of contraceptives and improving quality of birth spacing services are crucial to reduce unintended and unwanted pregnancies and abortions. Healthcare providers need to support PPD awareness with family planning counseling. Meanwhile, this study showed that the mothers who had no preterm birth history were more likely to be depressed than the counterpart mothers, different from the results of other studies in Nigeria (37) and Sweden (38) and one systematic review (17). Further studies are needed to investigate the association.

There was a positive association between antenatal depression experience and PPD in our study, despite no significance in multiple logistic models. British and Swedish studies showed that antenatal depression persists into the postnatal period in a large proportion of cases and many cases of PPD began in the antenatal period (39). The mothers who experienced antenatal depression had less likelihood to attend antenatal examinations which situation contributes to unfavourable pregnancy outcomes such as preterm delivery, low birth weight and poor neonatal outcomes. These findings could help identify women at risk of PPD (40). Identifying antenatal depression during pregnancy and referring high-risk women to appropriate healthcare providers can assist the diagnosis of antenatal depression and reduce the possibility of PPD.

In our study, mothers reporting they breastfed only was 78.2%, which is much higher than the national estimate (51.2%) (12). In Myanmar, the exclusive breastfeeding (EBF) rate has increased substantially from 23.6% in 2010 to 51.2% in 2016 and 90% of mothers were aware of breast milk benefits (41). Mixed feeding practice was more likely to be associated with PPD despite a marginal association in our study, supported by another online survey that women who were breastfeeding reported significantly better physical health and lower rates of depression than mixed-or formula-feeding mothers (42). Breastfeeding is affordable, helps with mother-infant bonding and has health benefits for both infant and mother. Moreover, breastfeeding mothers reported longer total sleep time, more daily energy and better physical health than their mixed-feeding counterparts (42). A qualitative study of Myanmar showed that returning to work was the main barrier to EBF and women with more knowledge about the benefits of breastfeeding had higher intentions to EBF (43). Thus, promotion of EBF by health staffs and media should be continued and it was suggested to advocate better support for breastfeeding in the workplace. Nonetheless, as this current study was conducted during the COVID-19 lockdown period, the higher prevalence of breastfeeding rate may suggest a need to study the association between infant feeding practice and the COVID-19 control measures as well as the concerns about COVID-19 infection.

### Associations with Factors Related to Health Services Utilisation

In addition, our study shows that 77.7% of mothers completed four times of ANC contacts and 96.8% made at least one PNC contact in our study, which is higher than those of national estimate (58.6% and 76.3%, respectively) (12). Furthermore, respondents who did not have ANC visits were four times more likely to be depressed compared to those who had completed ANC visits, this is in line with previous studies in Northwest Ethiopia (16) and Sudan (44). It may be associated with the care given during ANC visits, when counseling and anticipatory guidance is given by health providers to mothers. These kinds of care and services may build maternal self-esteem and resiliency, along with knowledge about pregnancy related problems and complications during visits.

The main reasons why mothers do not utilise ANC/PNC might be due to long travel time, inconvenient transportation and worries for travel cost even though they want to take MCH services. A sizable proportion of women, particularly in rural areas still find it difficult to access quality healthcare services. Our study shows that longer travel time (more than one hour) to health centres were more likely to be associated with PPD. Some studies had proved that longer distance and duration were directly associated with access to health services (45) due to less availability of healthcare providers, inadequacy of transport system and less timely care-seeking for health services. Patronage of health service is dependent on road conditions and access to a nearby health centre. Thus, more outreach visits such as mobile clinics, providing community health volunteers and health services planning by collaborating with local civil society organisations and authority will be required to reduce the barriers of accessibility.

### Associations with Social Support and Social Media Use

Our study shows that low social supports of husband and family are associated with PPD, although, these associations disappeared in multivariate analysis. These findings supported a previous study in Myanmar that low social support (husband, family and relatives) predisposed women to get PPD (8). The husband and family have an important role to play in the postpartum period because they are the closest person to support the newly fledged mother and their social support has been found to greatly influence the attitudes, emotions, and behaviors of new mothers (46). Another form of social support comes from social media. The role of social media in obtaining social support and health-related information and also promoting psychological well-being has been discussed in many studies. In our study, 38.2% of mothers used social media daily, while 86.8% and 44.5% of mothers used social media for seeking health information and emotional support, respectively. Mothers reported that suggestions and information from social media group (e.g. mother support group, blogging and online discussion) can make them feel comfortable and assist them in taking the right decision with regards to maternal and infant health. However, only 34.5% of the mothers in this study recommended social media usage to new mothers because they were annoyed by the impact of bad comments, unpleasant cases (accidents, child abuse, etc.) and missed information. Nonetheless, our study may provide some insights that social media might be a potential mediator to deliver health messages and knowledge, but usage pattern should be considered by comparing benefits and drawbacks.

### Limitations of the Study

The study has certain limitations when interpreting the findings of this study. Firstly, as our findings came from a cross-sectional survey, we cannot establish causality and could not measure the incidence of PPD. Secondly, as the study involves only postnatal mothers who can use and access online social media, the findings might not be representative of mothers in the township and Myanmar. Lastly, since this online survey was conducted over the COVID-19 lockdown period, the estimated prevalence of PPD seemed much higher. However, due to no other study on the prevalence of PPD during the lockdown period, this study is the first study to provide valuable information on the extent of PPD in LMICs.

## Conclusion

This study found that 31.8% of respondents had PPD and preterm delivery, pregnancy intention, breastfeeding status, birth interval and the frequency of ANC received and travel time to health centre were associated with PPD. The findings encouraged incorporating mental health services with existing MCH programme. The reasonable public health policy should promote PPD awareness and preventive interventions as well as reduce the barrier of health services accessibility to primary healthcare.

## Figures and Tables

**Table 1:** Distribution by sociodemographic characteristics and its associations with PPD among postpartum mothers

Variables name	Total	PPD		*P*-value

		No	Yes	

	*n* (%)	*n* (%)	*n* (%)	
	220 (100)	150 (68.2)	70 (31.8)	
Age of mothers (years old)				
< 25	57 (25.9)	38 (66.7)	19 (33.3)	
25–35	123 (55.9)	85 (69.1)	38 (30.9)	0.943
> 35	40 (18.2)	27 (67.5)	13 (32.5)	
Marital status				
Married and living with partner	212 (96.4)	148(69.8)	64(30.2)	0.008
Living without partner	8 (3.6)	2(25.0)	6(75.0)	
Education				
Under and complete middle school	107 (48.6)	69 (64.5)	38 (35.5)	0.252
Above middle school	113 (51.4)	81 (71.7)	32 (28.3)	
Current working status				
Working	78 (35.5)	48 (61.5)	30 (38.5)	0.117
Not working	142 (64.5)	102 (71.8)	40 (28.2)	
Occupation				
Waged/Employed	75 (34.1)	50(66.7)	25 (33.3)	0.729
Housewife	145 (65.9)	100 (69.0)	45 (31.0)	
Type of family				
Nuclear family	138 (62.7)	96 (69.6)	42 (30.4)	0.568
Extended family	82 (37.3)	54 (65.9)	28 (34.1)	
Monthly family income (MMK)				
< 200,000	110 (50.0)	66 (60.0)	44 (40.0)	0.021
200,000–400,000	80 (36.4)	59 (73.8)	21 (26.2)	
> 400,000	30 (13.6)	25 (83.3)	5 (16.7)	
Type of residence				
Urban	48 (21.8)	37 (77.1)	11 (22.9)	0.134
Rural	172 (78.2)	113 (65.7)	59 (34.3)	
Alcohol drinking				
Not at all	186 (84.5)	123 (66.1)	63 (33.9)	0.126
Yes	34 (15.5)	27 (79.4)	7 (20.6)	
Smoking				
Not at all	208 (94.5)	146 (70.2)	62(29.8)	0.008
Yes	12 (5.5)	4 (33.3)	8 (66.7)	
Betel quid chewing				
Not at all	169 (76.8)	125 (74.0)	44 (26.0)	0.001
Yes	51 (23.2)	25 (49.0)	26 (51.0)	

Note: MMK1 = USD0.00073

**Table 2:** Distribution by obstetric and infant factors and its associations with PPD among postpartum mothers

Variables name	Total	PPD		*P*-value

		No	Yes	

	*n* (%)	*n* (%)	*n* (%)	
Gravida				
Gravida 1	114 (51.8)	89 (78.1)	25 (21.9)	0.003
Gravida 2	52 (23.6)	32(61.5)	20 (38.5)	
Gravida 3, 4, 5	54 (24.5)	29 (53.7)	25 (46.3)	
Parity				
Primi parous	126 (57.3)	95 (75.4)	31 (24.6)	0.008
Multiparous	94 (42.7)	55 (58.5)	39 (41.5)	
Number of living children				
One child	128 (58.2)	96 (75.0)	32 (25.0)	0.010
More than one child	92 (41.8)	54 (58.7)	38 (41.3)	
Age of last child (month old)				
≤ 2	11 (5.0)	6 (54.5)	5 (45.5)	0.319
> 2	209 (95.0)	144 (68.9)	65 (31.1)	
Sex of last child				
Boy	116 (52.7)	75 (64.7)	41 (35.3)	0.236
Girl	104 (47.3)	75 (72.1)	29 (27.9)	
Preterm delivery (weeks)				
< 37	21 (9.5)	19 (90.5)	2 (9.5)	0.021
≥ 37	199 (90.5)	131 (65.8)	68 (34.2)	
Antenatal depression history				
Yes	25 (11.4)	11 (44.0)	14 (56.0)	0.006
No	195 (88.6)	139(71.3)	56 (28.7)	
Birth order				
First child	119 (54.1)	91 (76.5)	28 (23.5)	0.005
Second child	52 (23.6)	34 (65.4)	18 (34.6)	
Third or more	49 (22.3)	25 (51.0)	24 (49.0)	
Birth interval (years)				
< 2	150 (68.2)	106 (70.7)	44 (29.3)	0.071
2–5	45 (20.5)	32 (71.1)	13 (28.9)	
> 5	25 (11.4)	12 (48.0)	13 (52.0)	
Place of birth				
Home	38 (17.3)	21 (55.3)	17 (44.7)	0.040
Government health institution	151 (68.6)	103 (68.2)	48 (31.8)	
Private health institution	31 (14.1)	26 (83.9)	5 (16.1)	
Mode of delivery				
Vaginal	105 (47.7)	64 (61.0)	41 (39.0)	0.028
Caesarean	115 (52.3)	86 (74.8)	29 (25.2)	
Birth weight of the child (kg)				
≤ 2.5	190 (86.4)	132 (69.5)	58 (30.5)	0.301
< 2.5	30 (13.6)	18 (60.0)	12 (40.0)	
Pregnancy intention				
Planned	122 (55.5)	100 (82.0)	22 (18.0)	< 0.0001
Unplanned	98 (44.5)	50 (51.0)	48 (49.0)	
Breastfeeding status				
Breast milk only	172 (78.2)	125 (72.7)	47 (27.3)	0.007
Mixed feeding (breast milk and baby formula)	48 (22.0)	25(52.1)	23 (47.9)	
Supplementary feeding status				
Introduced less than 6 months	122 (55.5)	74 (60.7)	48 (39.3)	0.007
Introduced 6 months and later	98 (44.5)	76 (77.6)	22 (22.4)	

**Table 3:** Distribution by factors related to health centre utilisation and its associations with PPD among postpartum mothers

Variables name	Total	PPD		*P*-value

		No	Yes	

	*n* (%)	*n* (%)	*n* (%)	
Mode of travel to health centre				
By walk	38 (17.3)	29 (76.3)	9 (23.7)	0.237
By vehicles	182 (82.7)	121 (66.5)	61 (33.5)	
Time taken to reach health centre				
< 30 min	116 (52.7)	95 (81.9)	21 (18.1)	< 0.0001
30 min to 1 h	64 (29.1)	35 (54.7)	29 (45.3)	
> 1 h	40 (18.2)	20 (50.0)	20 (50.0)	
Travel cost				
≤ 5,000 MMK	188 (85.4)	130 (69.1)	58 (30.9)	0.455
>5,000 MMK	32 (14.5)	20 (62.5)	12 (37.5)	
Frequency of ANC received (four times)				
Not complete ANC visits	49 (22.3)	25 (51.0)	24 (49.0)	0.003
Complete ANC visits	171 (77.7)	125 (73.1)	46 (26.9)	
PNC services (at least one time)				
Yes	211 (95.9)	146 (69.2)	65 (30.8)	0.118
No	9 (4.1)	4 (44.4)	5 (55.6)	
PNC services (within 24 h of delivery)				
Yes	210 (95.5)	146 (69.5)	64 (30.5)	0.050
No	10 (4.5)	4 (40.0)	6 (60.0)	
Frequency of PNC received (four times)				
Not complete PNC visits	7 (3.2)	5 (71.4)	2 (28.6)	0.851
Complete PNC visits	213 (96.8)	145 (68.1)	68 (31.9)	

**Table 4:** Distribution by social support and social media use and its associations with PPD among postpartum mothers

Variables name	Total	PPD		*P*-value

		No	Yes	

	*n* (%)	*n* (%)	*n* (%)	
Husband support				
Low (1st tertile)	77 (35.0)	37 (48.1)	40 (51.9)	<0.0001
Moderate (2nd tertile)	92 (41.8)	72 (78.3)	20 (21.7)	
High (3rd tertile)	51 (23.2)	41 (80.4)	10 (19.6)	
Parent support				
Low (1st tertile)	78 (35.5)	41 (53.8)	36 (46.2)	0.003
Moderate (2nd tertile)	77 (35.0)	60 (77.9)	17 (22.1)	
High (3rd tertile)	65 (29.5)	48 (73.8)	17 (26.2)	
Family in law support				
Low (1st tertile)	74 (33.6)	43 (58.1)	31 (41.9)	0.070
Moderate (2nd tertile)	79 (35.9)	57 (72.2)	22 (27.8)	
High (3rd tertile)	67 (30.5)	50 (74.6)	17 (25.4)	
Relatives/Friends support				
Low (1st tertile)	79 (35.9)	54 (68.4)	25 (31.6)	0.202
Moderate (2nd tertile)	66 (30.0)	40 (60.6)	26 (39.4)	
High (3rd tertile)	75 (34.1)	56 (74.7)	19 (25.3)	
Frequency of usage of social media				
2–3 times a month	47 (21.4)	23 (48.9)	24 (51.1)	
1–3 times a week	89 (40.5)	62 (69.7)	27 (30.3)	0.003
Daily	84 (38.2)	65 (77.4)	19 (22.6)	
Social media usage for health information				
Yes	191 (86.8)	136 (71.2)	55 (28.8)	0.014
No	29 (13.2)	14 (48.3)	15 (51.7)	
Extent of health information support from social media				
Not at all	29 (13.2)	14 (48.3)	15 (51.7)	0.085
A little bit	44 (20.0)	31 (70.5)	13 (29.5)	
To some extent	84 (38.2)	62 (73.8)	22 (26.2)	
Very much	63 (28.6)	43 (68.3)	20 (31.7)	
Social media usage for emotional support				
Yes	98 (44.5)	65 (66.3)	33 (33.7)	0.596
No	122 (55.5)	85 (69.7)	37 (30.3)	
Extent of emotional support from social media				
Not at all	122 (55.5)	85 (69.7)	37 (30.3)	0.416
A little bit	26 (11.8)	20 (76.9)	6 (23.1)	
To some extent	57 (25.9)	37 (64.9)	20 (35.1)	
Very much	15 (6.8)	8 (53.3)	7 (46.7)	
Social media decreases stress and depressive symptoms				
Yes	102 (46.4)	79 (77.5)	23 (22.5)	0.006
No/Not sure	118 (53.6)	71 (60.2)	47 (39.8)	
Social media increases stress and depressive symptoms				
Yes	70 (31.8)	43 (61.4)	27 (38.6)	0.142
No/Not sure	150 (68.2)	107 (71.3)	43 (28.7)	
Mothers recommended for social media usage				
Yes	76 (34.5)	55 (72.4)	21 (27.6)	0.333
No/Not sure	144 (65.5)	95 (66.0)	49 (34.0)	

**Table 5:** Determinants of PPD among postpartum mothers

Variables name	Postpartum mothers (*n* = 220)		

	AOR	(95% CI)	*P*-value
Preterm delivery			
< 37 weeks	0.091	(0.013–0.650)	**0.017**
≥ 37 weeks	1		
Pregnancy intention			
Planned	1		
Unplanned	2.946	(1.301–6.670)	**0.010**
Breastfeeding status			
Breast milk only	1		
Mixed feeding (breast milk and baby formula)	2.347	(0.995–5.536)	0.051
Birth interval			
Less than 2 years	1		
2–5 years	0.679	(0.270–1.706)	0.410
More than 5 years	4.594	(1.637–12.891)	**0.004**
Frequency of ANC received			
Not complete ANC visits	2.518	(1.027–6.169)	**0.043**
Complete ANC visits	1		
Travel time to reach health centre			
Less than 30 min	1		
Within 30 min and 1 h	2.410	(1.044–5.565)	**0.039**
More than 1 h	3.068	(1.178–0.990)	**0.022**
Hosmer and Lemeshow Goodness of fit	11.509 (*P* = 0.174)		

Notes: Bold font indicates statistical significance (*P* < 0.05); Multiple logistic regression was used alongside the backward elimination method and variables included in the model were preterm delivery, pregnancy intention, breastfeeding, birth interval, frequency of ANC received, time taken to reach health centres, husband and parental support
